# Interaction between fasting blood glucose and tumor embolus in predicting the postoperative prognosis of 4330 Chinese patients with gastrointestinal tract cancer

**DOI:** 10.7150/jca.34843

**Published:** 2020-01-01

**Authors:** Dan Hu, Xinran Zhang, Xiandong Lin, Hejun Zhang, Yan Xia, Jinxiu Lin, Xiongwei Zheng, Feng Peng, Wenquan Niu

**Affiliations:** 1 Department of Pathology, Fujian Cancer Hospital & Fujian Medical University Cancer Hospital, Fuzhou, Fujian, China.; 2 Institute of Clinical Medical Sciences, China-Japan Friendship Hospital, Beijing, China.; 3 Department of Radiobiology, Fujian Cancer Hospital & Fujian Medical University Cancer Hospital, Fuzhou, Fujian, China.; 4 Department of Cardiology, The First Affiliated Hospital of Fujian Medical University, Fuzhou, Fujian, China.

**Keywords:** gastrointestinal tract cancer, tumor embolus, fasting blood glucose, interaction, prognosis, mortality

## Abstract

**Objectives:** We aimed to investigate the interaction between fasting blood glucose and tumor embolus, and the potential mediation effect of fasting blood glucose on tumor embolus in predicting gastrointestinal tract cancer-specific mortality risk postoperatively.

**Methods and Results:** 4330 patients were consecutively recruited between January 2000 and December 2010, with annual follow-up ending in December 2015. The median follow-up time was 48.6 months. Two optimal cutoff points for fasting blood glucose (6.11 and 11.69 mmol/L) were identified. Patients with fasting blood glucose <6.11 mmol/L and negative tumor embolus had the best survival, and the worst survival was seen in patients with fasting blood glucose >11.69 mmol/L and positive tumor embolus. The risk was highest for patients with fasting blood glucose >11.69 mmol/L and positive tumor embolus (adjusted HR: 11.91, 95% CI: 9.13 to 15.52). Using the Sobel-Goodman mediation test, the proportion of total effect conferred by tumor embolus that was mediated by fasting blood glucose was estimated to be 45.3%.

**Conclusions:** Our findings indicate a synergistic interaction between fasting blood glucose and tumor embolus in predicting the postoperative prognosis of gastrointestinal tract cancer.

## Introduction

Gastrointestinal tract cancer is a major cause of cancer-related morality worldwide, and it imposes a heavy burden on economies and public health 1,2. In China, the estimated incidence and mortality rates of gastrointestinal tract cancer in 2015 were separately 1055.4 and 689.0 per 100,000 persons 3. A major reason behind such high mortality is that a majority of cancer patients have advanced stages at diagnosis 4,5. Preventing or delaying the occurrence of cancer is no doubt important 6, but how to improve the short and long term prognosis and prolong life span is more important in current clinical practice.

We recently in the FIESTA cohort have analyzed a large panel of preoperative factors, and found that elevated fasting blood glucose was an independent and significant predictor for the poor prognosis of postoperative gastric and colorectal cancer, the two major components of gastrointestinal tract cancer 7,8. Both components are clinicopathologically heterogeneous, and tumor embolus is a fatal feature. Small tumor embolus is hard to detect because of nonspecific symptoms, and it often progresses rapidly 9. When tumor embolus is large enough, it often leads to ischemia or organ infarction, further participating a series of tumor pathophysiological complications 10,11. Given above lines of evidence, it would be tempting to speculate that fasting blood glucose might interact with or mediate the effect of tumor embolus on the mortality risk of gastrointestinal tract cancer. A literature search has, however, failed to reveal any evidence regarding this speculation.

To shed some light, we did an exploratory analysis of the FIESTA cohort patients to investigate the possible interaction between fasting blood glucose and tumor embolus, and the potential mediation effect of fasting blood glucose on tumor embolus at baseline in predicting the risk for gastrointestinal tract cancer-specific mortality postoperatively.

## Methods

### Study patients

All patients were consecutively recruited from the Fujian Provincial Cancer Hospital (the current Fujian Cancer Hospital & Fujian Medical University Cancer Hospital) during the period from January 2000 to December 2010, and they underwent the surgery. A total of 4330 postoperative patients with complete follow-up information and 25 to 87 years of age were incorporated in the current study. There were 3012 patients diagnosed with gastric cancer and 1318 patients diagnosed with colorectal cancer. All patients received no preoperative and postoperative chemotherapy or radiotherapy.

### Ethical approval

The conduct of the current study received approval from the ethical committees of Fujian Provincial Cancer Hospital, and each patient provided written informed consent prior to participation. The detailed design, eligibility criteria and follow-up evaluation have been described elsewhere 7,8.

### Baseline information

All patients were invited to complete a self-designed questionnaire to obtain demographic information at baseline, including age, gender, smoking status, drinking status and family cancer history. The recorded age was the age at the time of radical gastrectomy. Smoking status was categorized as never, former and current smoking, with the latter two combined as ever smoking. Drinking state was categorized as never, former and current alcohol drinking, with the latter two combined as ever drinking. The presence of family cancer history was recorded if one or more direct relatives are diagnosed with cancer (except non-melanoma skin cancer) within three generations. Body height and weight were taken on patients in light clothing and without shoes, and were used to calculate body mass index (BMI).

In the morning of surgery day, fasting (at least 8 hours) venous blood sample was collected into the EDTA-K2 anticoagulative tubes for measuring fasting blood glucose by the automated glucose oxidase method.

### Clinicopathologic information

Primary cancer and adjacent normal tissue biosamples were collected during surgery process, and they were fixed in 10% neutral-buffered formalin and further paraffin-embedded using standard procedures. Pathological assays were conducted at the Department of Pathology, Fujian Provincial Cancer Hospital. Clinicopathologic features such as tumor-node-metastasis (TNM) stage (I, II, III and IV) and tumor embolus that were diagnosed according to the 7^th^ Edition of the UICC/AJCC TNM Staging system 12 were extracted from pathological reports.

### Follow-up evaluation

Patients were interviewed every six to twelve months by face-to-face interview or by phone calls or postal mails if they missed appointments at the Out-Patient Department, Fujian Provincial Cancer Hospital. The follow-up began from the first recruitment after the surgery since January 2000 to the date of death due to causes other than gastric or colorectal cancer or the end of patient follow-up visit in December 2015, whichever came first. The primary outcome was death from gastric or colorectal cancer.

### Statistical analysis

The optimal fasting blood glucose was selected through survival tree analysis (STREE program, http://c2s2.yale.edu/software/stree/) developed by Professor Heping Zhang 13. The survival tree analysis is constructed on the basis of recursive partitioning algorithms. The root node in a survival tree represents a sample of study participants where the tree is grown-learning sample, and an offspring node uses the same splitting factor as its ancestors. Once the tress is saturated, the recursive partitioning process stops, meaning that offspring nodes cannot be further split.

Kaplan-Meier survival function and log-rank test were used to depict and compare cumulative survival rates across groups by fasting blood glucose and tumor embolus. A restricted cubic spline regression model was fitted to delineate the association between fasting blood glucose and gastrointestinal tract cancer-specific mortality by tumor embolus status using STATA software, version 14.1 (StataCorp, TX, USA). Proportional hazards assumption was judged by weighted Schoenfeld residuals. Hazard ratio (HR) and 95% confidence interval (95% CI) were derived under the Cox proportional regression model. The Sobel-Goodman mediation test 14 was employed to test whether fasting blood glucose carried the influence of tumor embolus on the primary outcome. A mediator is a variable that accounts for all or part of the relation between a predictor and an outcome, and mediational analysis is useful in studies where several mediators are targeted by an experimental manipulation 15. The power of detecting significant prediction was estimated using the PS (Power and Sample Size Calculations) software, version 3.0.

## Results

### Baseline characteristics

Table 1 provides the characteristics of cohort patients with gastrointestinal tract cancer at baseline. The follow-up period ranged from 1.1 months to 188.9 months, with a median follow-up time of 48.6 months. At the end of follow-up (December 2015), 1743 deaths from either gastric or colorectal cancer occurred, leaving 2587 survivors in the analysis. Positive tumor embolus presented in a significantly higher percentage in non-survivors than in survivors (48.14% versus 26.28%, P<0.001).

### Optimal cutoff determination

**Figure 1:**
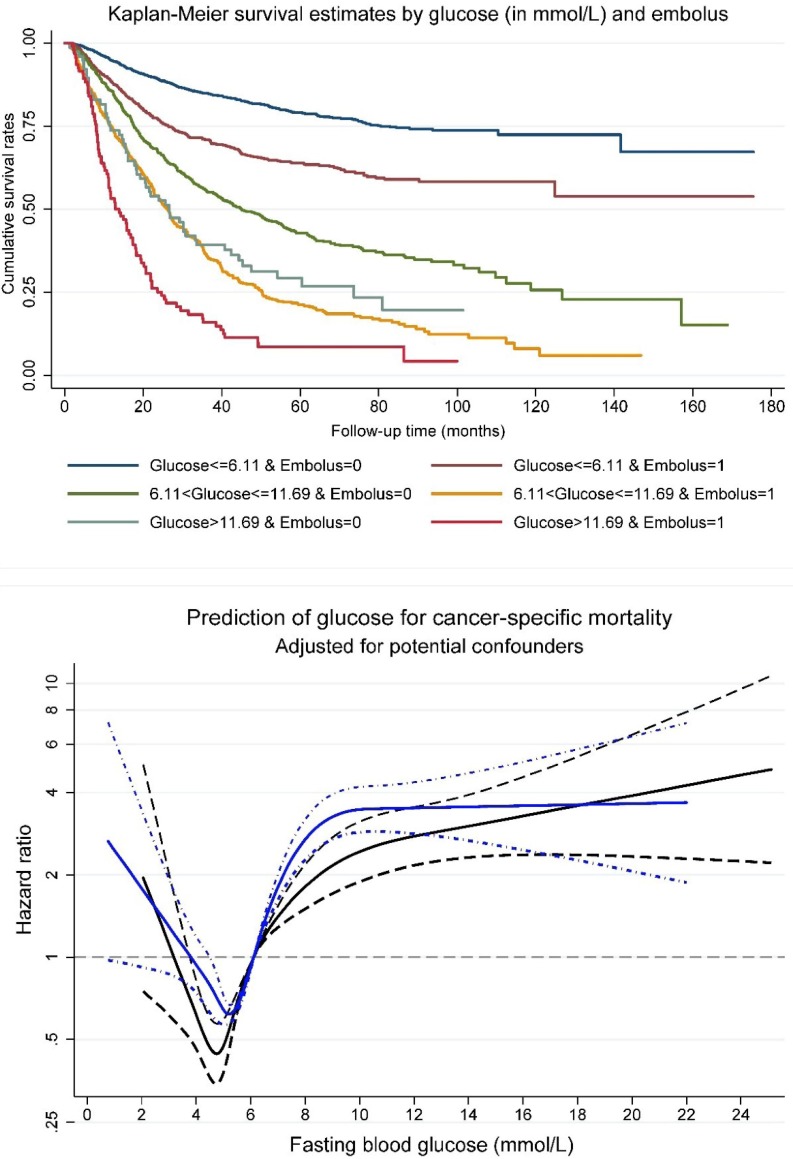
Kaplan-Meier survival curve per fasting glucose (in three groups) and tumor embolus in combination (upper panel), and association between fasting glucose and cancer-specific mortality risk (lower panel) among patients with gastrointestinal tract cancer. In lower panel, navy line was plotted for positive embolus, and blue line for negative embolus. Dotted lines represent 95% confidence intervals.

Survival tree analysis identified two optimal cutoff points for fasting blood glucose (6.11 and 11.69 mmol/L) that can split patients with distinct survival rates. A combination of fasting blood glucose at the two cutoff points and tumor embolus status was generated to split all study patients into six non-overlapped groups. The upper panel of Figure 1 shows cumulative survival rates of above six groups. Patients with fasting blood glucose less than 6.11 mmol/L and negative tumor embolus had the best survival, and by contrast, the worst survival was seen in patients with fasting blood glucose greater than 11.69 mmol/L and positive tumor embolus.

### Prognosis prediction

The restricted cubic spline regression model indicated that patients with positive tumor embolus had a poorer postoperative prognosis than those with negative tumor embolus with the increase of fasting blood glucose (Figure 1, the lower panel).

**Figure 2:**
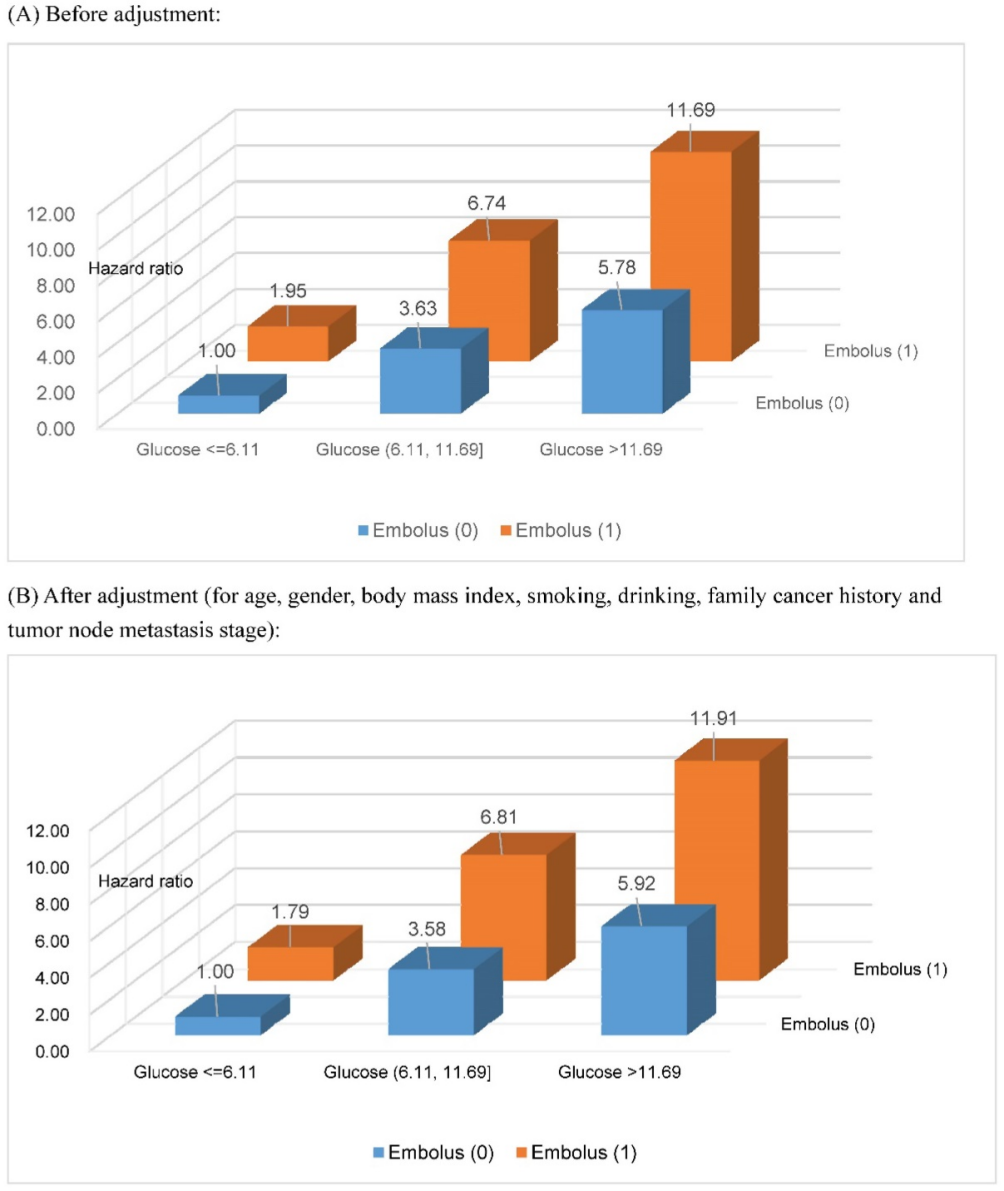
Risk estimation of fasting blood glucose and tumor embolus in combination for gastrointestinal tract cancer-specific mortality. The unit for fasting blood glucose is mmol/L. All risk estimates were significant at a level of 0.001.

Based on the two optimal cutoff points of fasting blood glucose (6.11 and 11.69 mmol/L) selected by survival tree analysis, together with tumor embolus status, all study patients were divided into six groups. After assigning patients with fasting blood glucose ≤6.11 mmol/L and negative tumor embolus as a reference group, the risk estimates for gastrointestinal tract cancer-specific mortality are delineated in Figure 2 with and without adjusting for potential confounders, including age, gender, BMI, smoking status, drinking status, family cancer history and TNM stage, and all estimates were significantly higher than the unity (P<0.001). Overall, there was an increasing trend in risk estimates with the increase of fasting blood glucose in patients with both negative and positive tumor embolus, and the effect was more obvious in patients with positive tumor embolus. As expected, the risk was highest for patients with fasting blood glucose >11.69 mmol/L and positive embolus (adjusted HR: 11.91, 95% CI: 9.13 to 15.52). The power to detect significance when estimating the risk for gastrointestinal tract cancer-specific mortality was over 95% for above comparisons.

In addition, for the risk prediction of fasting blood glucose and tumor embolus in combination for gastrointestinal tract cancer-specific mortality, robust permutation testing was performed using 1000 bootstrap replications subsequently, and no material changes in effect-size estimates were noticed (data not shown).

### Mediation test

The mediation test indicated that fasting blood glucose was a qualified mediator to the extent to which it carried the influence of tumor embolus on the primary outcome, as fasting blood glucose differed significantly between patients with positive (mean ± standard deviation: 6.56 ± 2.74 mmol/L) and negative (5.96 ± 2.26 mmol/L, P<0.001) tumor embolus, tumor embolus was associated with the significant mortality risk in patients with normal fasting blood glucose (<6.1 mmol/L according to the criteria set forth by the Chinese Diabetes Society in 2004 16) (HR: 1.78, 95% CI: 1.50 to 2.12, P<0.001), fasting blood glucose was independently associated with the mortality risk (per fasting glucose increment: HR: 1.20, 95% CI: 1.19 to 1.22, P<0.001), and the effect of tumor embolus on mortality risk shrink upon the addition of fasting blood glucose to the Cox proportional regression model (HR: 2.08 versus 1.93, 95% CI: 1.87 to 2.31 versus 1.74 to 2.15). According the results of the Sobel-Goodman mediation test, the proportion of total effect conferred by tumor embolus that was mediated by fasting blood glucose was estimated to be 45.3%, indicating that nearly half the effect of tumor embolus on gastrointestinal tract cancer-specific mortality was accounted by fasting blood glucose.

## Discussion

Via an exploratory analysis of 4330 postoperative patients after a median follow-up of 48.6 months, the novel findings reported here are that there is a synergistic interaction between fasting blood glucose and tumor embolus at baseline in predicting the prognosis of gastrointestinal tract cancer. More specifically, fasting blood glucose may serve as a potent mediator and account for about half of effect of tumor embolus on mortality risk. To the best of our knowledge, this is the first study that has interrogated the relationship between fasting glucose and tumor embolus in postoperative patients with gastrointestinal tract cancer.

It is widely recognized that patients with hyperglycemia or diabetes mellitus had an increased risk of developing thromboembolism, as well as in cancer patients 17-19. However, data are particularly sparse on the implication of hyperglycemia or diabetes mellitus in the formation and prognosis of tumor embolus. To fill this gap in knowledge, we explored the relation between fasting blood glucose and tumor embolus, and observed that occurrence of high glucose and positive embolus was exponentially and remarkably associated with an enhanced risk of gastrointestinal tract cancer-specific mortality, indicating a possible synergistic interaction between fasting blood glucose and tumor embolus. Further, we found that fasting blood glucose satisfied the prerequisites of a qualified modifier and nearly half of the effect of tumor embolus was mediated through fasting blood glucose, giving rise to speculation that elevated blood glucose might be a robust harbinger of tumor embolus progression in postoperative patients with gastrointestinal tract cancer. Thus far, the underlying mechanisms whereby blood glucose mediated the prognostic impact of tumor embolus are not yet fully understood. It is possible that elevated glucose could infract vascular endothelium that is the primary defense against thrombosis and enhanced activation of platelets and clotting factors, consequently resulting in plasma hypercoagulability 20-24. On the other hand, plasma clots in patients with elevated glucose exhibited reduced permeability and a more compact fibrin network structure, with smaller pore sizes, increased fiber thickness and number of branch points as characteristics 25,26. What's more, elevated glucose may slow fibrinolysis rates as a result of reducing plasminogen binding to the fibrin network coupled with impaired plasmin generation 26. Irrespective of the mechanisms, confirmation of our findings in other large, long follow-up cohort populations will be important for clarifying the relation between blood glucose and tumor embolus in the prognostic prediction of gastrointestinal tract cancer.

Another important finding of the current study was the identification of optimal fasting blood glucose cutoff points at baseline that can help group patients with distinct prognostic outcomes. According to the criteria set forth by the Chinese Diabetes Society in 2004 16, fasting blood glucose equal to and greater than 6.1 mmol/L is defined as hyperglycemia. In this study, we selected two optimal cutoff points for fasting blood glucose by means of survival tree analysis, and one point was 6.11 mmol/L, in close proximity to the hyperglycemia diagnostic cutoff. We additionally identified another higher cutoff point, 11.69 mmol/L, for fasting blood glucose, and we importantly observed that patients with fasting blood glucose greater than 11.69 mmol/L and positive tumor embolus were over ten times more likely to die of gastric or colorectal cancer than those with fasting glucose less than 6.11 mmol/L and negative tumor embolus. This finding is of clinical importance as we, on the basis of fasting glucose and tumor embolus at baseline, can inform future risk assessment, formulate cancer management strategies and prioritize rational planning of medical resources in patients with gastrointestinal tract cancer.

A few limitations of the current study should be acknowledged. First, this study was carried out in a mono center, and we agree that external validations of our findings are necessary and important. In addition, all study patients were of Han Chinese descent from south China, and thus the generalizability may be limited. Second, we measured fasting blood glucose and tumor embolus only at the time of recruitment, and their changes after the surgery would be more valuable. Third, data on glycated hemoglobin were not available, and it would be more informative if glycated hemoglobin was included in the analysis. Fourth, only cancer-specific mortality was recorded, and other clinical outcomes such as recurrence and progression-free survival are not available for analysis.

In conclusion, our findings indicate a synergistic interaction between fasting blood glucose and tumor embolus at baseline in predicting the prognosis of gastrointestinal tract cancer. Specifically, fasting blood glucose may serve as a potent mediator and account for about half of effect of tumor embolus on mortality risk. The implication of this epidemiological study is clinically relevant with respect to primary and secondary prevention of tumor embolus, and controlling fasting blood glucose in postoperative patients with gastrointestinal tract cancer will improve the prognosis of tumor embolus.

## Figures and Tables

**Table 1:** Baseline characteristics of cohort patients with gastrointestinal tract cancer.

Variables	Survivors (n=2235)	Non-survivors (n=1558)	P
Age (years)	57.38 (11.53)	58.60 (12.09)	<0.001
Sex (male)	69.31%	69.65%	0.811
Ever smoking	17.41%	16.90%	0.675
Ever drinking	4.95%	5.09%	0.838
Family cancer history	8.75%	8.67%	0.928
Body mass index (kg/m^2^)	22.77 (3.00)	23.05 (3.19)	0.006
Fasting blood glucose (mmol/L)	5.41 (1.63)	7.15 (2.97)	<0.001
Tumor node metastasis stage			<0.001
I	20.37%	2.30%	
II	28.61%	10.74%	
III	49.14%	57.50%	
IV	1.88%	29.46%	
Tumor embolus	26.28%	48.14%	<0.001

Data are expressed as mean (standard deviation) or number (percentage) where appropriate. *P was calculated using the Mann-Whitney test or the Chi-square test when appropriate.
